# Health-Related Effects of the Elderly Care Program

**DOI:** 10.1155/2018/7121037

**Published:** 2018-05-20

**Authors:** Young-Eun Kim, Seok-Won Hong

**Affiliations:** ^1^National Evidence-Based Healthcare Collaborating Agency (NECA), Seoul, Republic of Korea; ^2^Department of Preventive Medicine, College of Medicine, Korea University, Seoul, Republic of Korea

## Abstract

**Objectives:**

This study aimed to determine the health-related effects of elderly care through the elderly program (ECEP) compared to those who were put on standby.

**Methods:**

Analysis was conducted concerning the demographic characteristics and chronic disease prevalence status of 108,803 ECEP participants from 2007 to 2013 and 33,932 recipients of elderly care by the elderly (ECE) in 2013. A survey was conducted to determine the effects of emotional support on 508 elders who received ECE in 2014. To determine the effect of elderly care by participation, a comparative analysis was performed using the propensity score method and a differences-in-differences model. Statistical tests were performed on these study subjects' medical expenses and utilization of medical care, while they participated in the ECEP.

**Results:**

ECEP participants incurred a lower total medical expense increase by US$431.94, US$75.54 lower copayment, US$357.60 lower insurance payment, and US$403.04 lower hospitalization costs, compared to the elders who were on standby. Furthermore, a significant decrease was observed in the number of days of hospitalization, even in the case of medical care utilization. Those who were receiving elderly care scored an average of 7.70 points on the Short Geriatric Depression Scale. 51% of these individuals showed a significantly high level of depression, with a score of 8 points or higher, which is a criterion for screening for depression.

**Conclusions:**

The present study showed differences in medical expenses and the utilization of medical care associated with ECEP.

## 1. Background

South Korea became an aging society in 2000, with 7.2% of its population being aged ≥ 65 years. In August 2017, more than 14% of the population comprised the elderly population and entered the aging society. The country's aging population is rapidly progressing, compared to those of other countries such as Japan, where aging had already progressed. A decrease in the working-age population and labor productivity is expected to accompany the progression of the aging society. Thus, the government began promoting a senior employment program in 2004, as a comprehensive solution for elderly issues, due to the increase in social problems such as the retirement of the baby-boomer generation and elderly poverty and suicide rates. The senior employment program aims to save social costs such as health maintenance and supplemental income support and to promote social participation.

In 2005, the ECEP was implemented as part of a senior employment program. The ECEP is a program in which healthy elders manage or support the daily lives of other vulnerable elders with limited mobility. The ECEP can be defined as an activity that eliminates blind spots in the welfare system and improves welfare recipients' quality of life. In the ECEP, healthy elders provide recipients with emotional support, such as checking their health status, talking to them, reading books to them, and helping them with housekeeping chores such as laundry, cooking, and shopping. The health and medical support provided includes support with regard to taking medication and accompanying vulnerable elderly people to the hospital, the doctor's office, and the pharmacy. Not only does the ECEP provide supplementary income by providing work to those who need it but also it provides welfare services to recipients by matching them with elderly care providers.

The health-related effect of elderly labor has been proven in previous studies. Many studies have found elderly labor to effectively improve health due to nonfinancial compensation such as self-realization, increased self-esteem, recognition of participation as a member of society, and social support [1–7]. Studies have also shown association between income and elderly's health [5, 8–10]. This means that poverty, indicative of the inability to sufficiently meet basic needs such as food, clothing, and shelter, may have a negative effect on health [9, 10]. However, because elderly labor increases household income and reduces financial difficulties, it has a positive effect on elders' physical health [8]. It was found that working elders show an interest in health-related information in order to keep their jobs, practice caution while working, take care of their health during their spare time, utilize health support systems, and make an effort to establish a healthy lifestyle [11] and that such health maintenance behaviors had positive effects on their health.

Research has also shown the negative effects of elderly labor on health. A study has shown that, because their bodily functions are vulnerable due to aging, the health and safety of working elderly people may be threatened and, thus, working in later years could negatively affect their health [12]. Furthermore, working in old age may have negative effects on health in that although elders may retain their jobs, the recurrence rate of arthritis, diabetes, and high blood pressure is high, and such diseases may threaten their functioning in daily life [13].

As seen in previous studies, it is known that working in old age has both positive and negative effects on health [1–13]. Accordingly, the present study attempted to quantitatively determine the effect of elderly people's participation in the ECEP on health; the ECEP was implemented so as to create work for the elderly in South Korea. The study sought to identify changes in medical expenses and utilization of medical care or differences between ECEP participants and recipients. In addition, the study entailed an evaluation of the effect in terms of emotional support of ECEP on elderly beneficiaries who received services through the ECEP.

## 2. Methods

To determine current participation in the ECEP and the characteristics of the participating and benefiting elders, analysis was conducted on the demographic characteristics and chronic disease prevalence status of 108,803 ECEP participants of 2007 to 2013 and on 33,932 ECE recipients of 2013. Forty-six chronic diseases suggested by Van Den Bussche et al. [14], such as cancer, hypertension, osteoarthrosis, diabetes mellitus, and depression, were considered.

In addition to the above, the effect of emotional support was investigated among 508 elderly who received elderly care in 2014 by surveying satisfaction regarding the ECE service, subjective health improvement effects after receiving the service, and depressive emotion. For depression, we used the short form of the Geriatric Depression Scale-Korean (SGDS-K) [15], which is a Korean version of a 15-item, abridged version of a 30-item self-report depression scale developed by Yesavage and colleagues. And the effect of subjective health improvement was investigated through two questionnaires. The health status of the participants before and after their participation in the ECEP was evaluated on a 5-point scale. The survey population was 31,477 elderly persons receiving elderly care services at 523 ECEP implementation organizations nationwide in 2014. To secure the representativeness of the sample, sampling was carried out through the stratified cluster sampling method, using regions as strata and participating organizations as clusters. The survey was conducted through face-to-face interviews by surveyors trained from 25 August to 30 September 2014.

To determine the effect of participation in the elderly care program, a comparative analysis was performed on medical expenses and the utilization of medical care by 12,412 elderly persons who had participated in the ECEP for more than three months in 2013 and by the 3,307 seniors who were on standby for program participation who wished to participate in the welfare type senior employment program in 2013. A combined analysis was performed on the data of program participants and the people on standby for the senior employment program and the health insurance claims data and health insurance eligibility data received from the National Health Insurance Service (NHIS). To analyze differences in changes in medical expenses and the utilization of medical care according to participation in the ECEP, the differences-in-differences model was utilized. To strengthen the comparability between groups according to participation in the ECEP, the inverse probability weight proposed by Rosenbaum and Rubin [16], using the propensity score, was applied. The differences-in-differences model is a type of quasi-experimental research method used in estimating the causal effects of an intervention. This method entails comparing the differences in the periods before and after an intervention within a group influenced by the intervention from the exogenous intervention with similar differences within a nonintervention group [17].

It is important to set up a comparison group to confirm the effect of an intervention through the differences-in-differences model. An ideal comparison group should satisfy the condition that all characteristics other than participation in the ECEP are identical (parallel trend). However, because it is practically impossible to randomly assign participation in the ECEP, subjects making up the comparison group were selected using the next best method, which is choosing those who wanted to but did not participate in the ECEP. Those who did not participate in the ECEP can be classified as people who were unqualified, who had not applied, who withdrew application, who were excluded from selection, and who were on standby. According to Lee [18], the unqualified and those who had not applied were inappropriate, as they had a high possibility of having heterogeneous characteristics, compared to those who participated in the program. Moreover, those who were excluded and were on standby had significantly similar characteristics to those who participated in the program in unobservable characteristics that could cause selection bias when compared. Therefore, the present study attempted to secure as much homogeneity as possible in all characteristics other than participation in ECEP, using elders who participated in the ECEP and those who applied to participate in the ECEP but were on standby and expected to have similar characteristics other than participation itself as the comparison group [18–20]. The present study applied the inverse probability weight model using the propensity score to strengthen comparability between the groups, according to participation. The balance between the ECEP participating group and nonparticipating group was confirmed, using the standardized difference. When the standardized difference was smaller than 0.1, the applicable covariates are judged as balanced [21–23].

To identify differences in changes in medical expenses and utilization of medical care according to participation in the ECEP, differences between the two groups were analyzed using the generalized linear model, with the medical expenses and utilization of medical care during 2012 and 2013 as the dependent variables. For medical expenses, total medical costs, pharmaceutical costs, outpatient costs, and hospitalization costs were analyzed. For utilization of medical care, days under medical care, days of hospitalization, the number of days as an outpatient, and the number of medication prescription days were analyzed.

## 3. Results

### 3.1. Characteristics of Participants and Recipients of the ECEP

It was found that the average participation period of 108,803 elderly persons who participated in the ECEP more than once, between 2007 and 2013, was about 7.1 months. Characteristics of elders who participated in the ECEP are illustrated below. There were more female (83.08%) than male participants; most were aged 70–79 years (56.90%), followed by the group aged 65–69 years (37.94%) and the 80–89-year-old age group (5.10%). With regard to household characteristics, the majority (42.86%) of ECEP participants lived alone, 29.32% were living as an elderly couple, 17.57% lived with a family member with financial means, and 6.28% lived with a family member without financial means. An analysis of the prevalence of chronic diseases among participating elders showed an average of 3.80 illnesses (Table 1).

**Figure 1:**
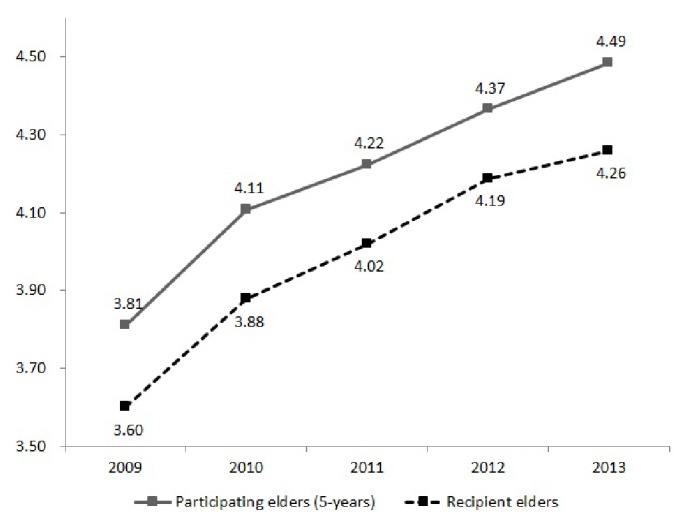
Number of chronic diseases among participants in the elderly care by the elderly program.

To indirectly determine the healthy-worker effect on participation in the ECEP, the number of chronic illnesses was compared between elders who had participated in the program for five years in a row, from 2009 to 2013, and elders on standby. If there is a healthy-worker effect, then those who had participated for five years in a row should be healthier than the elderly on standby. The results showed that the number of chronic diseases among elderly persons who had participated in the program for five years in a row was higher than that of elders on standby, which meant that there was no healthy-worker effect (Figure 1).

It was found that, among the 33,932 recipients of elderly care in 2013, 81.88% were female and 18.12% were male. Summarizing by age, 80–89-year-olds made up 43.79%, 70–79–year-olds made up 43.58%, ≥90-year-olds made up 6.63%, and <70-year-olds made up 6.00%. The mean age was 79.48 years (SD = 6.77 years). Summarizing by household composition, 83.30% of the elderly were living alone, followed by those living as an elderly couple, at 4.66%. There were 4.01 chronic diseases among recipients of elderly care (Table 1).

### 3.2. Service Satisfaction among Recipients of the ECE Service and the Effects of Emotional Support

The results showed that 89.89% of the elderly care recipients were satisfied with the ECE service, 8.53% reported average satisfaction, and 1.58% were dissatisfied. On a scale of 1 to 7 (“extremely dissatisfied” to “extremely satisfied,” resp.) the mean score was 5.87 (SD = 1.08). In terms of age, recipients in their 60s and below obtained a mean score of 5.63; those in their 70s, a mean score of 5.77, those in their 80s, a mean score of 5.92, and those aged ≥ 90 years, 6.05. This indicated higher satisfaction regarding the ECEP according to age. Differences in satisfaction per age group were statistically significant (*p* < .0001, table is not shown).

To determine the effect of the elderly care service on subjective health improvement, a survey was conducted on the subjective health condition before and during the reception of elderly care service. The rating scale ranged from 1 to 5. The results showed that the subjective health condition before receiving the service was represented by a mean score of 2.72 but was at 3.00 while the elderly were receiving the service. The average change in the subjective health condition before and after reception of the service was 0.28 points, which was a statistically significant change (*p* < .0001; Table 2).

A mean score of 7.70 (SD = 4.62) was obtained for the short form of the Geriatric Depression Scale (S-GDS), which measured elderly depression. The S-GDS uses an 8-point scale for depression screening [24]. For elderly participants receiving ECE, more than 51.0% obtained a score of ≥8.

### 3.3. Differences in Changes in Medical Expenses and Utilization of Medical Care according to Participation in the ECEP

To analyze differences in changes in medical expenses and utilization of medical care according to participation in the ECEP in 2013, the characteristics of ECEP participants and the elderly on standby were compared. The two groups were nonequivalent, with the exception of residence type. After applying inverse probability weight using the propensity score to obtain comparability between ECEP participants and the elderly on standby, equivalence between the groups was checked. It was found that, in all categories of all covariates, the standardized difference values were less than 0.1, which showed that covariates were balanced (Table 3).

The analysis of differences in the increase in medical expenses according to participation in the ECEP showed that the total increase in the medical expenses of ECEP participants was US$ 431.94 less than that of the elderly on standby; this difference was statistically significant (*p* < .0001). The increase in the medical expenses on copayment, insurance payment, and hospitalization costs incurred by ECEP participants was less than that of the standby group by US$ 75.54, US$ 357.60, and US$ 403.04, respectively; all were statistically significant (*p* < .0001). There were no differences between the two groups in outpatient and medication costs (Table 4).

**Figure 2:**
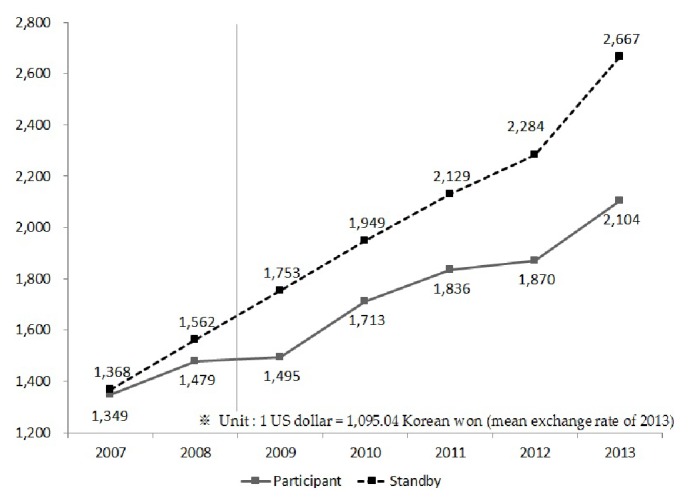
Trend of medical costs among elders on standby and those who participated in the program for five years in a row.

To determine the effect of long-term ECEP participation, the total expenses of medical care incurred by elderly persons who had participated in the ECEP for five years in a row (2009–2013) and elderly persons on standby were compared. There was almost no difference in the total amount of medical care expenses in 2007 and 2008 when both groups did not participate in the program. However, from 2009, when participation commenced, the differences increased. The difference in medical expenses was US$ 82.33 in 2008, just before participation in the program, but increased to US$ 257.14 in 2009 once the program had started, with the difference at US$ 562.75 in 2013 (Figure 2).

An analysis on the utilization of medical care showed that the increased number of hospitalization days among ECEP participants was 2.24 days less than that of the standby group; this difference was statistically significant (*p* < .0001). There were no differences between the groups with regard to changes in medical care days, outpatient days, and medication prescription days (Table 4).

## 4. Discussion

The present study sought to determine the characteristics of “participating elders” who acquired a job and “recipient elders” who received services through the ECEP, which was first implemented in 2007. We aimed to determine what program effect resulted from participation in the ECEP and benefits in terms of health effects. A total of 108,803 elderly persons participated in the ECEP from 2007 and 2013; participants were mostly women and mean of age was increased. Compared to elderly persons on standby, ECEP participants incurred an increase in total medical expenses which was US$ 431.94 less, US$ 75.54 less for copayment, US$ 357.60 less for insurance payment, and US$ 403.04 less for hospitalization costs. Furthermore, with regard to medical care utilization, a significant decrease was observed in the number of days of hospitalization for the participating group.

According to the most recently conducted study by Lee et al. [25], the total medical cost savings of the welfare type labor program, which includes the ECEP, forming part of senior employment programs, were US$ 434.44; this is very similar to the total medical cost savings of US$ 431.94 of the ECEP in the present study. It is, however, difficult to interpret such results as indicative of improvements in the level of health, as a result of participation in the ECEP and its accompanying effects of a decrease in medical expenses and utilization of medical care. This is because it would not be feasible to measure changes in individuals' health condition before and after participation in the ECEP. The findings of the present study are significant, as health-related effects due to participation in the ECEP were indirectly determined. Accordingly, research is required on the effect of the ECEP on improvements in health, a decrease in medical expenses, utilization of medical care, and quality of life, by directly measuring aspects such as ECEP participants' health and quality of life.

Even though this study found differences in the increment of medical expenses and utilization of medical care, according to participation in the ECEP, evaluation of the benefits of ECEP in terms of economic aspects should be avoided. This is because participation in the ECEP makes immeasurable social contributions, such as recovering one's self-esteem as an active member of society and reducing the sense of alienation.

The short form of the Geriatric Depression Scale (S-GDS) yielded an average score of 7.70 among ECE recipients; 51.0% of this group showed significantly high levels of depression, with a score of 8 or higher, which is a criterion during screening for depression. Evidently, it is necessary to establish an intimately connected system with local medical and welfare-related resources to strengthen the emotional support provided to recipients of ECE services and to reduce depression among the elderly.

The significance of the present study is its evaluation of the effects of the ECEP, with a focus on medical expenses and the utilization of medical care, as indicators of elderly people's health. Not only does the ECEP provide elderly persons with jobs but also it provides welfare services to recipients by matching them with elderly care providers. Therefore, the effects of emotional support on emotions such as depression and a sense of alienation can be expected to manifest through emotional exchanges between the ECEP participants and recipients.

The present study empirically showed differences in medical expenses and the utilization of medical care, based on participation in ECE. However, the study has a limitation in that it did not determine whether or not the effect on medical expenses and medical care utilization was directly due to participation in the program. Although the results were obtained with the selection bias maximally controlled for, there is a limitation in the analysis as to the circumstances under which ECEP participation hampers the observed differences in medical expenses. Actually, ECEP participation effectively suppresses use of medical care due to time constraints, while also increasing the use of necessary medical care, due to an increase in income. It is necessary to find out whether the difference in medical expenses according to ECEP participation is a positive effect resulting from improvement in health or suppression of use of medical care due to time constraints. Considering the fact that participation in ECEP takes 36–40 working hours per month (two days per week), the reasoning that the difference in medical expenses is due to limited use of medical care due to time constraint seems somewhat improbable. Nevertheless, future research in this regard could identify circumstances under which differences in changes in medical expenses and utilization of medical care occur.

It is the hope of the current investigators that the ECEP in aging South Korea can be proposed as an alternative to solving the problems of the aged by identifying the potential effects through analysis of the long-term health of elderly participants in the ECEP in the future.

## 5. Conclusions

The present study showed differences in the increase in medical expenses and the utilization of medical care based on participation in ECE. The significance of the present study is its evaluation of the effects of the ECEP, with a focus on medical expenses and the utilization of medical care, as an indicator of elderly people's health. Not only does the ECEP provide elderly persons with jobs, but also it provides welfare services to recipients by matching them with elderly care providers. Therefore, the effects of emotional support on such things as depression and feelings of alienation can be expected to manifest through emotional exchanges between the ECEP participants and care recipients. However, this study has a limitation in that it did not determine whether the effect on medical expenses and medical care utilization was directly due to participation in the program. Although the results were obtained while selection bias was being controlled for as much as possible, the analysis is limited as to the circumstances under which ECEP participation hampers the observed differences in medical expenses.

## Figures and Tables

**Table 1:** Characteristics of participants and beneficiaries of the elderly care by the elderly program.

Classification	Participating elderly ('07–'13)	Recipient elderly ('13)
*N*	108,803	33,932
Gender (%)		
Male	16.92	18.12
Female	83.08	81.88
Age group (%)		
65–69 years	37.94	6.00
70–79 years	56.90	43.58
80–89 years	5.10	43.79
≥90 years	0.06	6.63
Type of household (%)		
Living alone	42.86	83.30
Elderly couple	29.32	4.66
Elderly couple with children	1.12	0.00
Family living together (no financial means)	6.28	2.18
Family living together (have financial means)	17.57	1.72
Others	3.84	3.03
Number of chronic diseases (mean ± SD)	3.80 ± 1.82	4.01 ± 2.65

**Table 2:** Subjective health improvement effects among recipients of the elderly care by the elderly program.

Before		After		Difference (after−before)		
Mean	SD	Mean	SD	Mean	SD	*p*value^*∗*^
2.72	0.8851	3.00	0.7839	0.28	0.8045	<.0001

^*∗*^Paired  *t*-test.

**Table 3:** A comparison of general characteristics of elderly program participants and standby elderly in 2013 before and after the application of Inverse Probability of Treatment Weighted.

Parameter	Before IPTW application			After IPTW application		
	Participating elderly	Standby elderly	*p*value^*∗*^	Participating elderly (*n* = 15,289)	Standby elderly (*n* = 15,496)	Standardized difference
Gender						
Male	16.42	31.25	<.0001	19.48	18.81	0.017
Female	83.58	68.75		80.52	81.19	−0.017
Age category						
<70 years	26.35	29.12		7.46	7.99	−0.020
70–79 years	66.20	63.44	0.0053	65.70	65.30	0.008
80 years or older	7.45	7.44		26.84	26.71	0.003
Household composition type						
Living alone	44.46	30.66		41.68	42.74	−0.021
Elderly couple	27.28	32.99		28.32	27.31	0.023
Family living together (no financial means)	7.00	6.86	<.0001	6.99	6.75	0.009
Family living together (have financial means)	19.63	27.64		21.59	21.64	−0.001
Others	1.63	1.84		1.41	1.55	−0.012
Medical security type						
Medical benefits	0.76	2.30	<.0001	1.09	1.12	−0.003
Medical insurance	99.24	97.70		98.91	98.88	0.003
Education level						
No education	25.19	22.71		24.70	24.50	0.005
Elementary school	49.01	44.72		48.08	48.32	−0.005
Middle school	15.41	18.82	<.0001	16.09	16.14	−0.001
High school	8.44	11.05		9.01	8.98	0.001
College or above	1.94	2.69		2.12	2.06	0.004
Health condition						
Bad	3.49	5.90		4.01	4.06	−0.003
Average	45.18	44.57	<.0001	44.80	45.02	−0.004
Good	51.33	49.53		51.19	50.92	0.005
Financial condition						
Low	52.66	54.57		53.28	53.33	−0.001
Average	44.49	42.11	0.0302	43.80	43.68	0.002
High	2.85	3.31		2.92	2.99	−0.004
Residence type						
Monthly rent	10.92	10.38	0.1975	10.83	11.30	−0.015
Lease	13.92	13.64		13.93	14.57	−0.018
Own home	67.19	66.92		67.01	65.94	0.023
Others	7.97	9.06		8.23	8.19	0.001
Implementing agency						
Seniors Welfare Center	30.04	29.91		30.04	29.18	0.019
Social Welfare Center	13.25	21.65		14.98	14.56	0.012
The Korean Senior Citizens Association	12.45	8.01		11.74	12.82	−0.033
Seniors Welfare Center	13.22	6.80	<.0001	11.81	11.66	0.005
Local government	12.61	7.44		11.25	12.03	0.024
Senior club	11.17	16.81		12.40	11.95	0.014
Others	7.28	9.37		7.78	7.80	−0.001

^*∗*^Chi-squared test.

**Table 4:** Difference in medical expense changes and utilization of medical care changes according to participation in the elderly care by the elderly program.

Classification	Estimation^*∗*^	SE	95% confidence limits		*p* value
			Lower	Upper	
Difference in medical expense changes (unit: 1 US dollar^*∗∗*^)					
Total medical expenses	−431.94	95.86	−580.63	−283.25	<.0001
Copayment	−75.54	12.95	−100.93	−50.16	<.0001
Insurance payment	−357.60	65.46	−485.90	−229.30	<.0001
Outpatient costs	−29.04	16.71	−61.80	3.72	0.0823
Hospitalization costs	−403.04	69.73	−539.71	−266.36	<.0001
Medication costs	0.25	8.24	−15.90	16.39	0.9760

Difference in utilization of medical care changes (unit: days)					
Medical care days	−6.19	3.25	−12.56	0.17	0.0566
Hospitalization days	−2.24	0.37	−2.96	−1.52	<.0001
Outpatient days	−0.57	0.54	−1.64	0.49	0.2934
Medication prescription days	−3.23	2.93	−8.97	2.51	0.2701

^*∗*^Gender, age, family composition, medical insurance type, level of education, subjective health condition, subjective financial status, residence type, implementing agency, number of chronic diseases, and number of surgeries adjusted for. ^*∗∗*^1 US dollar = 1,095.04 Korean won (mean exchange rate of 2013).

## Data Availability

The data that support the findings of this study are available from NECA and NHIS in Korea but restrictions apply to the availability of these data, which were used under license for the current study and so are not publicly available. Data are, however, available from the authors upon reasonable request and with permission of NECA and NHIS.

## Abbreviations

ECEP: Elderly care through the elderly program
ECE: Elderly care by the elderly
S-GDS: Short form of the Geriatric Depression Scale.
